# Administration of Harmine and Imipramine Alters Creatine Kinase and Mitochondrial Respiratory Chain Activities in the Rat Brain

**DOI:** 10.1155/2012/987397

**Published:** 2011-09-29

**Authors:** Gislaine Z. Réus, Roberto B. Stringari, Cinara L. Gonçalves, Giselli Scaini, Milena Carvalho-Silva, Gabriela C. Jeremias, Isabela C. Jeremias, Gabriela K. Ferreira, Emílio L. Streck, Jaime E. Hallak, Antônio W. Zuardi, José A. Crippa, João Quevedo

**Affiliations:** ^1^Laboratório de Neurociências and Instituto Nacional de Ciência e Tecnologia Translacional em Medicina (INCT-TM), Programa de Pós-Graduação em Ciências da Saúde, Unidade Acadêmica de Ciências da Saúde, Universidade do Extremo Sul Catarinense, 88806-000 Criciúma, SC, Brazil; ^2^Laboratório de Bioenergética and Instituto Nacional de Ciência e Tecnologia Translacional em Medicina (INCT-TM), Programa de Pós-Graduação em Ciências da Saúde, Unidade Acadêmica de Ciências da Saúde, Universidade do Extremo Sul Catarinense, 88806-000 Criciúma, SC, Brazil; ^3^Departamento de Neurociências e Ciências do Comportamento and Instituto Nacional de Ciência e Tecnologia Translacional em Medicina (INCT-TM), Faculdade de Medicina de Ribeirão Preto, Universidade de São Paulo, 14049-900 Ribeirão Preto, SP, Brazil

## Abstract

The present study evaluated mitochondrial respiratory chain and creatine kinase activities after administration of harmine (5, 10, and 15 mg/kg) and imipramine (10, 20, and 30 mg/kg) in rat brain. After acute treatment occurred an increase of creatine kinase in the prefrontal with imipramine (20 and 30 mg/kg) and harmine in all doses, in the striatum with imipramine (20 and 30 mg/kg) and harmine (5 and 10 mg/kg); harmine (15 mg/kg) decreased creatine kinase. In the chronic treatment occurred an increase of creatine kinase with imipramine (20 mg/kg), harmine (5 mg/kg) in the prefrontal with imipramine (20 and 30 mg/kg) and harmine (5 and 10 mg/kg) in the striatum. In the acute treatment, the complex I increased in the prefrontal with harmine (15 mg/kg) and in the striatum with harmine (10 mg/kg); the complex II decreased with imipramine (20 and 30 mg/kg) in the striatum; the complex IV increased with imipramine (30 mg/kg) in the striatum. In the chronic treatment, the complex I increased with harmine (5 mg/kg) in the prefrontal; the complex II increased with imipramine (20 mg/kg) in the prefrontal; the complex IV increased with harmine (5 mg/kg) in the striatum. Finally, these findings further support the hypothesis that harmine and imipramine could be involved in mitochondrial function.

## 1. Introduction

For more than 30 years, it has been theorized that levels of monoamines, such as serotonin, norepinephrine, and dopamine, are generally low in the brain during untreated major depressive episodes [1]. In fact, the clinically used antidepressants increase the extracellular concentrations of monoamines, serotonin, or norepinephrine either by inhibiting their reuptake from the synapse or by blocking their degradation by inhibiting monoamine oxidase [2–4].

The *β*-carboline harmine is present in plants, such as *Peganum harmala *and *Banisteriopsis caap*, which are used for ritual and medicinal preparations [5]. Also, ingestion of Ayahuasca, which contains harmine in its composition improved psychometric measures of panic and hopelessness in humans [6]. Recently, a growing body of evidence has indicated that harmine presents antidepressant-like actions in rodents subjected to an animal model of depression [7–10]. In addition, studies have demonstrated that harmine interacts with monoamine oxidase A (MAO-A) [11] and several cell-surface receptors, including serotonin receptor 2A (5-HT2A) [12], which are involved in antidepressant pharmacotherapy [13].

The creatine/phosphocreatine/CK system is important for normal to maintain the energy homeostasis [14, 15] exerting several integrated functions, such as temporary energy buffering, metabolic capacity, energy transfer, and metabolic control [16]. The brain of adult rats, like other tissues with high and variable rates of ATP metabolism, presents high phosphocreatine concentration and creatine kinase activity [14, 15]. 

Also important for cellular energy metabolism are the mitochondria, which are organelles that play a crucial role in ATP production, a process carried out by the respiratory chain complexes I, II, III and VI [17]. Besides, studies have indicated that impairment in the energy metabolism may be involved in pathophysiology of some neuropsychiatric disorders, such as bipolar disorder and major depression [18–20]. In fact, our group recently demonstrated that rats submitted to the chronic mild stress presented an increased oxidative stress in submitochondrial particles in the brain [19]. Moreover, we also demonstrated a reduction in creatine kinase and citrate synthase activities in brain of rats submitted to the animal model of mania [21, 22]. We recently also showed that mitochondrial respiratory chain complexes I, II-III, and IV were inhibited after chronic mild stress in cerebral cortex and cerebellum and acute administration of ketamine reversed the complexes inhibition [20]. In addition, some studies have appointed to a role of *β*-carbolines in energy metabolism [21] and in oxidative stress [23, 24], which is involved with mitochondrial function. Also, there is also a relationship between mitochondria and monoamines. In fact, we observed decreased levels of serotonin in brain of mouse with multiple mtDNA deletions [25].

Thus, the main objective of our study was to assess the effects induced by acute and chronic administration of *β*-carboline harmine and tricyclic antidepressant imipramine on the energy metabolism in the brain of rats by evaluation of mitochondrial respiratory chain (complexes I, II, II-III, and IV) and creatine kinase activities.

## 2. Results

**Figure 1:**
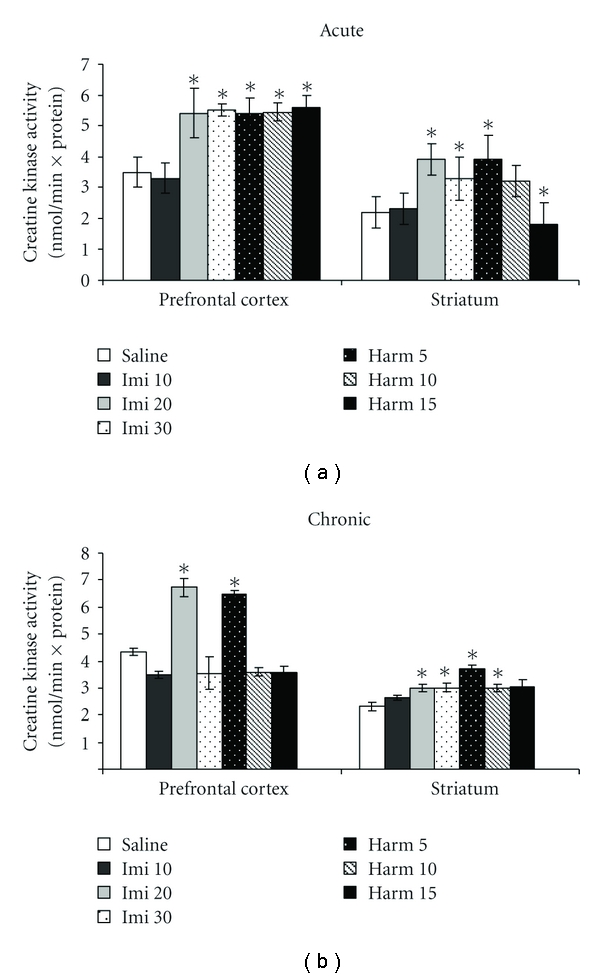
Effects of the acute (a) and chronic (b) administration of harmine (5, 10, and 15 mg/kg, i.p.) and imipramine (10, 20, and 30 mg/kg, i.p.) on creatine kinase activity in the prefrontal cortex and striatum of rats. Bars represent means ± SEM of 6 rats. *P < 0.05 versus saline according to ANOVA followed by Tukey post hoc test.

Acute administration of harmine at the doses of 5, 10, and 15 mg/kg and imipramine at the doses of 20 and 30 mg/kg increased creatine kinase activity in the prefrontal cortex (ANOVA; *P* < 0.05; Figure 1(a)). In the striatum, harmine at the dose of 5 mg/kg and imipramine at the doses of 20 and 30 mg/kg increased the creatine kinase activity; however, harmine at the dose of 15 mg/kg decreased creatine kinase activity in the striatum (ANOVA; *P* < 0.05; Figure 1(a)). In the chronic treatment with imipramine at the dose of 20 mg/kg and harmine at the dose of 5 mg/kg occurred an increase in creatine kinase activity in the prefrontal cortex, compared to control group (ANOVA; *P* < 0.05; Figure 1(b)). In the striatum chronic treatment with imipramine at the doses of 20 and 30 mg/kg and harmine at doses of 5 and 10 mg/kg increased the creatine kinase activity, compared to control group (ANOVA; *P* < 0.05; Figure 1(b)).

**Figure 2:**
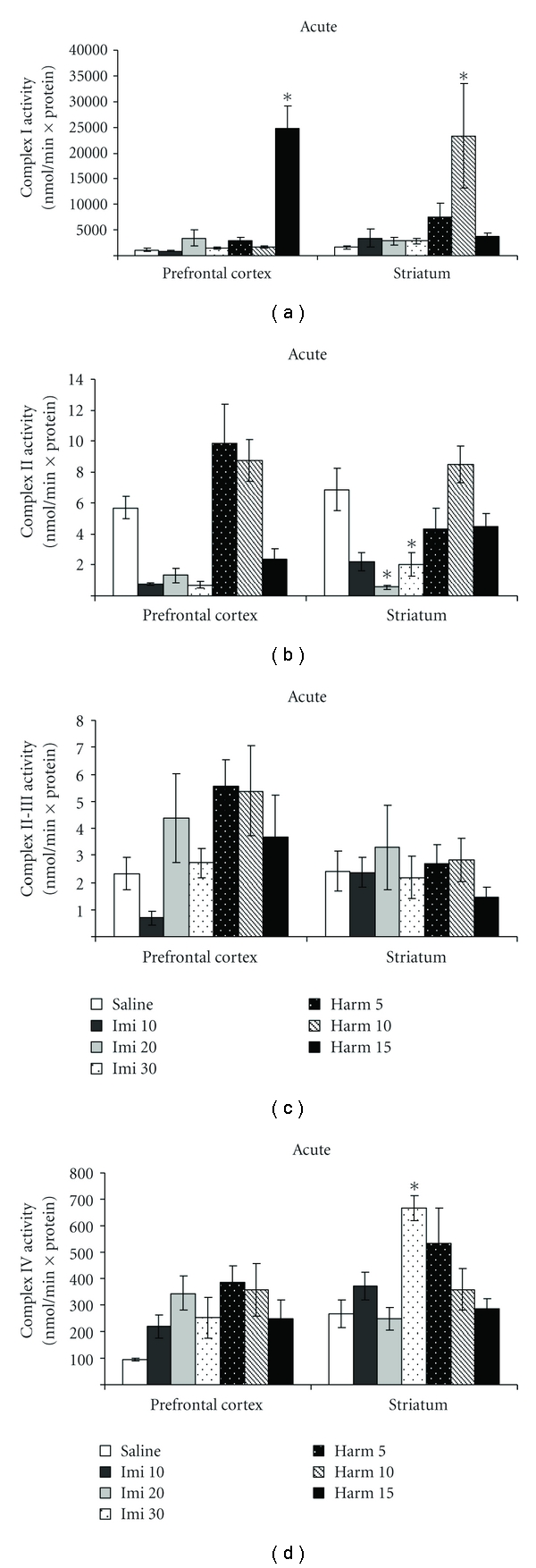
Effects of the acute administration of harmine (5, 10 and 15 mg/kg, i.p.) and imipramine (10, 20 and 30 mg/kg, i.p.) on complex I (a), II (b), II-III (c), and IV (d) activities in the prefrontal cortex and striatum of rats. Bars represent means ± SEM of 6 rats. *P < 0.05 versus saline according to ANOVA followed by Tukey post hoc test.

Figure 2 shows the mitochondrial respiratory chain (I, II, II-III, and IV) after acute treatment with imipramine and harmine. The complex I activity increased in prefrontal cortex after acute treatment of harmine at the dose of 15 mg/kg, and in striatum after treatment with harmine at the dose of 10 mg/kg (ANOVA; *P* < 0.05; Figure 2(a)); acute treatment with imipramine did not alter complex I activity in rat brain. The complex II activity was decreased after acute treatment with imipramine at the doses of 20 and 30 mg/kg in striatum (ANOVA; *P* < 0.05; Figure 2(b)); harmine did not alter complex II activity in both prefrontal cortex and striatum. The complex II-III activity did not alter after acute treatment with imipramine or harmine (ANOVA; *P* > 0.05; Figure 2(c)). The complex IV activity increased only in striatum after acute treatment with imipramine at the dose of 30 mg/kg (ANOVA; *P* < 0.05; Figure 2(d)).

**Figure 3:**
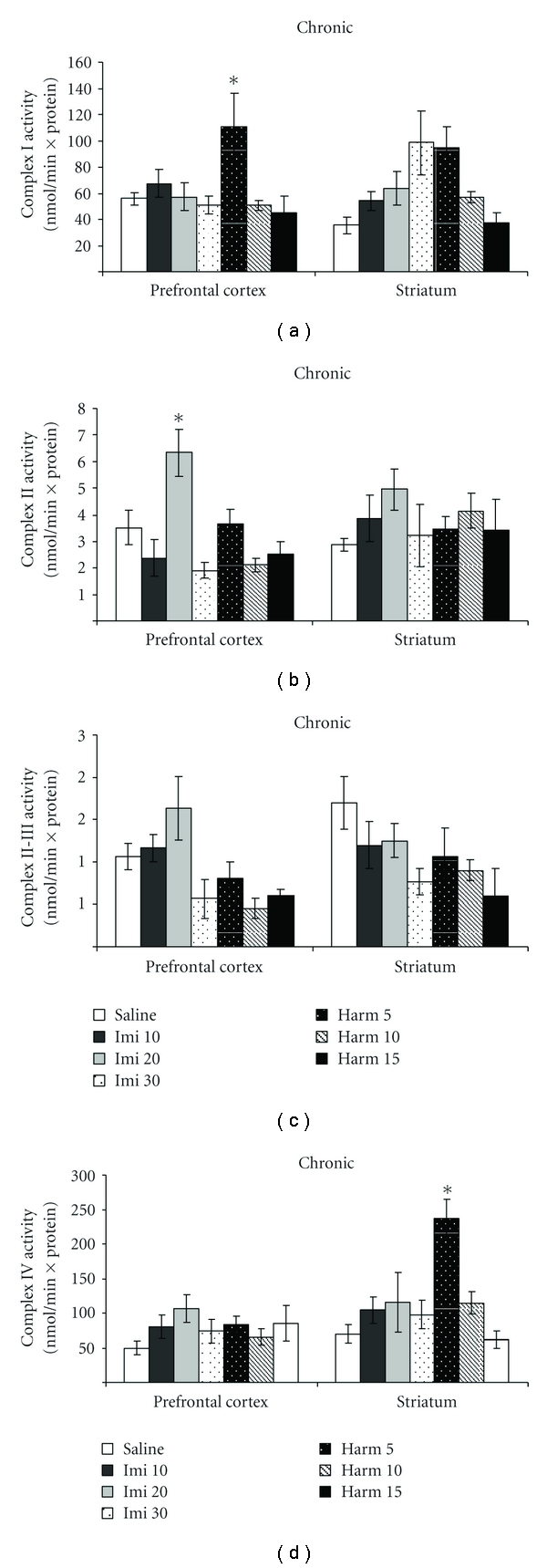
Effects of the chronic administration of harmine (5, 10, and 15 mg/kg, i.p.) and imipramine (10, 20, and 30 mg/kg, i.p.) on complex I (a), II (b), II-III (c), and IV (d) activities in the prefrontal cortex and striatum of rats. Bars represent means ± SEM of 6 rats. *P < 0.05 versus saline according to ANOVA followed by Tukey post hoc test.

In the chronic treatment with harmine at the dose of 5 mg/kg occurred an increase in the complex I activity in prefrontal cortex (ANOVA; *P* < 0.05; Figure 3(a)), and in the complex IV activity in striatum (ANOVA; *P* < 0.05; Figure 3(d)), compared to control group. The complex II activity increased only in prefrontal cortex with imipramine at the dose of 20 mg/kg (ANOVA; *P* < 0.05; Figure 3(b)). The complex II-III activity did not alter with chronic treatment with imipramine or harmine (ANOVA; *P* > 0.05; Figure 3(c)).

## 3. Discussion

In the present study we demonstrated that acute and chronic treatments with harmine or imipramine altered respiratory chain complexes and creatine kinase activities in the rat brain. However, we observed that these alterations were dependent on treatment regime, complex, brain area, and drug concentration.

Studies from our group recently showed that acute treatment with harmine and imipramine decreased immobility time of rats and increased both climbing and swimming time of rats; in addition harmine, but not imipramine, increased BDNF (brain-derived neurotrophic factor) protein levels in the rat hippocampus [9, 10]. Nevertheless, harmine reverted the behavioral and physiological parameters caused to chronic mild stress model [6]. Additionally, Farzin and Mansouri [7] demonstrated that treatment with harmane, norharmane, and harmine dose dependently reduced the immobility time in the mouse. 

Damage to the mitochondrial electron transport chain has been suggested to be an important factor in the pathogenesis of a range of psychiatric disorders [26–28], including major depression. In fact, Gardner et al. [29] showed a significant decrease in mitochondrial ATP production rates and mitochondrial enzyme ratios in muscles of major depressive disorder patients. Madrigal et al. [27] also reported that complexes I-III and II-III of mitochondrial respiratory chain were inhibited in rat brain after chronic stress by immobilization. Our group also recently demonstrated that mitochondrial respiratory chain was inhibited in brain of rats after chronic variable stress, suggesting that energy metabolism impairment may occur in depressive disorders [20].

Our findings in the present data showed the effects of the *β*-carboline harmine and imipramine on the creatine kinase activity in the prefrontal cortex and striatum; the brain areas were used because they are important in psychiatric diseases. The prefrontal cortex is directly involved in emotion and cognition and thereby contributes to other major symptoms of mood disorders [30]. In addition, the striatum is a dopaminergic area involved to memory [31] and mood disorders [32].

Our group recently also showed that imipramine increased creatine kinase activity in the striatum, cerebral cortex, cerebellum and prefrontal cortex and did not alter creatine kinase activity in the hippocampus [33]. In another study was showed that acute and chronic treatments with fluoxetine decreased the creatine kinase activity in the rat brain [34], suggesting that the inhibition of creatine kinase activity by these drugs may be associated to some effects of fluoxetine.

 In this present data we showed that acute and chronic treatments with harmine or imipramine altered complex I, II and VI activities in the prefrontal cortex or striatum, but not complex II-III activities in both treatments. We do not know how to explain why this effect occurred, but we suggest that the nonactivation of complex II-III might be a harmine mechanism of action, possibly harmine leads to a selective increase of individual mitochondrial respiratory chain enzyme activities. 

The effects of antidepressants in the energy metabolism have been demonstrated. In fact, prolonged *in vivo* imipramine treatment sustained stimulation of respiratory activity, accompanied by increased cytochromes aa_3_ and c + c1 content; in addition, the content of cytochrome aa_3 _ increased steadily up to the second week of treatment and of cytochrome c + c_1 _ steadied state level by the end of the first week [35]. Additionally, an *in vitro* study from pig brain, using different antidepressants and mood stabilizers, showed a decrease of I, II, and IV complexes activity [36]. In this study the authors suggest that these mitochondrial enzymes may be candidates in searching for new biological markers of mood disorders, targets of new antidepressants, or predictors of response to pharmacotherapy. In addition, antidepressant treatment inhibited monoamine oxidase A and B in the mitochondrial fraction of rat brain [37]. Thus, the effects of harmine on the energy metabolism in the present study may be related, at least in part, to its action in the monoamine oxidase or serotonin. In fact, harmine interacts with MAO-A [11] and 5-HT2A [12], and, interestingly, decrease in serotonin levels was related with mtDNA deletions [25], thus showing a relationship with monoamines and mitochondria.

The effects of harmine and imipramine in the present study may also be related to oxidative stress. In fact, it is well known that the reactive oxygen species cause damage in the mitochondrial oxidative phosphorylation system and that reactive oxygen species itself is vulnerable to the mitochondrial dysfunction [38, 39]. Very recently we demonstrated that both acute and chronic treatments with imipramine and harmine reduced lipid and protein oxidation and increased superoxide dismutase and catalase in the rat prefrontal cortex and hippocampus, suggesting positive effects of imipramine antidepressant and harmine in oxidative stress parameters [24]. Additionally, Zafir et al. [40] demonstrated that treatment with some antidepressants recovered the activities of superoxide dismutase, catalase, glutathione S-transferase, glutathione reductase, glutathione and normalized lipid peroxidation product malondialdehyde, and protein carbonyl following stress-induced restraint in rodents.

Moreover, the *β*-carbolines, harmaline and harmalol, and the antioxidants (superoxide dismutase, catalase, ascorbate, or rutin) prevented the loss of cell viability in PC12 cells treated with methyl-4-phenylpyridinium (MPP^+^); in addition, harmaline and harmalol reduced the condensation and fragmentation of nuclei and inhibited the decrease in mitochondrial transmembrane potential, cytochrome c release, activation of caspase-3, formation of reactive oxygen species, and depletion of glutathione caused by MPP^+^ in PC12 cells [23], suggesting a protective effect of *β*-carboline on neural cells. In contrast, other study showed that harmine elicits cytotoxicity via mitochondrial dysfunction [41], demonstrating that exposure of cell culture hepatocytes to harmine causes a concentration and time-dependent decrease in cell viability and losses of intracellular ATP, adenine nucleotides, glutathione and protein thiols, and an increase in glutathione disulfide and reactive oxygen species.

In this study we suggested that the action mechanism of imipramine antidepressant and *β*-carboline harmine may be, at least in part, involved in the creatine kinase and mitochondrial respiratory chain activities, but these effects were dose and treatment relatedin rat brain areas. In conclusion, considering that metabolism impairment is probably involved in the pathophysiology of depressive disorders, the modulation of energy metabolism by antidepressants could be an important mechanism of action of these drugs and harmine could be studied as a new drug for the treatment of depression.

## 4. Materials and Methods

### 4.1. Animals

Male Adult Wistar rats (60 days old) were obtained from UNESC (Universidade do Extremo Sul Catarinense, Criciúma, SC, Brazil) breeding colony. They were housed five per cage with food and water available *ad libitum* and were maintained on a 12 h light/dark cycle (lights on at 7:00 AM). All experimental procedures involving animals were performed in accordance with the NIH Guide for the Care and Use of Laboratory Animals and the Brazilian Society for Neuroscience and Behavior (SBNeC) recommendations for animal care and with approval by local Ethics Committee under protocol number 325/2008.

### 4.2. Drugs and Treatments

Harmine was obtained from THC-Pharm/STI-Pharm (Frankfurt, Germany) and imipramine, the standard antidepressant, from Novartis Pharmaceutical Industry (São Paulo, Brazil). Different groups of rats (*n* = 6 each) were administrated intraperitoneally (i.p.) with saline, (control group) or different doses of harmine (5, 10, and 15 mg/kg) or imipramine (10, 20, and 30 mg/kg) one single time (acute treatment) or during 14 days once a day (chronic treatment) [9, 10]. All treatments were administered in a volume of 1 mL/kg. Two hours after last injection in both acute and chronic treatments, all rats were killed by decapitation, and the skulls, prefrontal cortex, and striatum were removed.

### 4.3. Tissue and Homogenate Preparation

Prefrontal cortex and striatum were homogenized (1 : 10, w/v) in SETH buffer, pH 7.4 (250 mM sucrose, 2 mM EDTA, 10 mM Trizma base, 50 IU/mL heparin).

### 4.4. Creatine Kinase Activity

The homogenates were centrifuged at 800 ×g for 10 min. and the supernatants were kept at −70°C until used for creatine kinase activity determination. The maximal period between homogenate preparation and enzyme analysis was always less than 5 days. Protein content was determined by the method described by Lowry et al. [42], using bovine serum albumin as standard. Creatine kinase activity was measured in brain homogenates pretreated with 0.625 mM lauryl maltoside. The reaction mixture consisted of 60 mM Tris-HCl, pH 7.5, containing 7 mM phosphocreatine, 9 mM MgSO_4_, and approximately 0.4–1.2 *μ*g protein in a final volume of 100 *μ*L. After 15 min. of preincubation at 37°C, the reaction was started by the addition of 0.3 *μ*moL of ADP plus 0.08 *μ*moL of reduced glutathione. The reaction was stopped after 10 min. by the addition of 1 *μ*moL of hydroxymercuribenzoic acid. The creatine formed was estimated according to the colorimetric method [43]. The colour was developed by the addition of 100 *μ*L 2%  *α*-naphthol and 100 *μ*L 0.05% diacetyl in a final volume of 1 mL and read spectrophotometrically after 20 minutes at 540 nm. Results were expressed as nmoL/m × mg protein.

### 4.5. Respiratory Chain Enzyme Activities

The homogenates were centrifuged at 800 ×g for 10 min and the supernatants were kept at –70°C until used for enzyme activity determination. The maximal period between homogenate preparation and enzyme analysis was always less than 5 days. Protein content was determined by the method described by Lowry et al. [42] using bovine serum albumin as standard. NADH dehydrogenase (complex I) was evaluated by the method described by Cassina and Radi [44] by the rate of NADH-dependent ferricyanide reduction at 420 nm. The activity of succinate: Cytochrome *c *oxidoreductase (complexes II-III) was determined according to the method of Fischer et al. [45], measured by Cytochrome *c *reduction from succinate. The activity of Cytochrome *c *oxidase (complex IV) was assayed according to the method described by Rustin et al. [46], measured by following the decrease in absorbance due to the oxidation of previously reduced Cytochrome *c *at 550 nm. The activities of the mitochondrial respiratory chain complexes were expressed as nmoL/min ×mg  protein.

### 4.6. Statistical Analysis

All data are presented as mean ± SEM. Differences among experimental groups in the assessment of mitochondrial respiratory chain and creatine kinase activities were determined by one-way ANOVA, followed by Tukey post hoc test when ANOVA was significant; *P* < 0.05 was considered to be statistically significant.
